# Crystal structure of poly[bis­(μ_2_-5-hydroxy­nicotinato-κ^2^ *N*:*O* ^3^)zinc]

**DOI:** 10.1107/S2056989015000249

**Published:** 2015-01-14

**Authors:** Wen-Bing Wang, Shan-Shan Xu, Hong-Ji Chen

**Affiliations:** a Department of Material Science and Engineering, Jinan University, Guangzhou 510632, People’s Republic of China

**Keywords:** crystal structure, zinc coordination polymer, 5-hy­droxy­nicotinate ligand, hydrogen bonding

## Abstract

The title coordination polymer, [Zn(C_6_H_4_NO_3_)_2_]_*n*_, was prepared under hydro­thermal conditions by the reaction of zinc nitrate with 5-hy­droxy­nicotinic acid in the presence of malonic acid. In the structure, the Zn^II^ ion is coordinated by two carboxyl­ate O atoms and two pyridine N atoms of four 5-hy­droxy­nicotinate ligands in a distorted tetra­hedral coordin­ation environment. The μ_2_-bridging mode of each anion leads to the formation of a three-dimensional framework structure. Inter­molecular hydrogen bonds between the hy­droxy groups of one anion and the non-coordinating carboxyl­ate O atoms of neighbouring anions consolidate the crystal packing.

## Related literature   

For transition metal complexes with 5-hy­droxy­nicotinate ligands, see: Jiang & Feng (2008 ▸); Zhang *et al.* (2011 ▸); Yang *et al.* (2010 ▸). For corresponding rare earth metal complexes, see: Zhang *et al.* (2012 ▸); Mi *et al.* (2012 ▸); Xu *et al.* (2013 ▸).
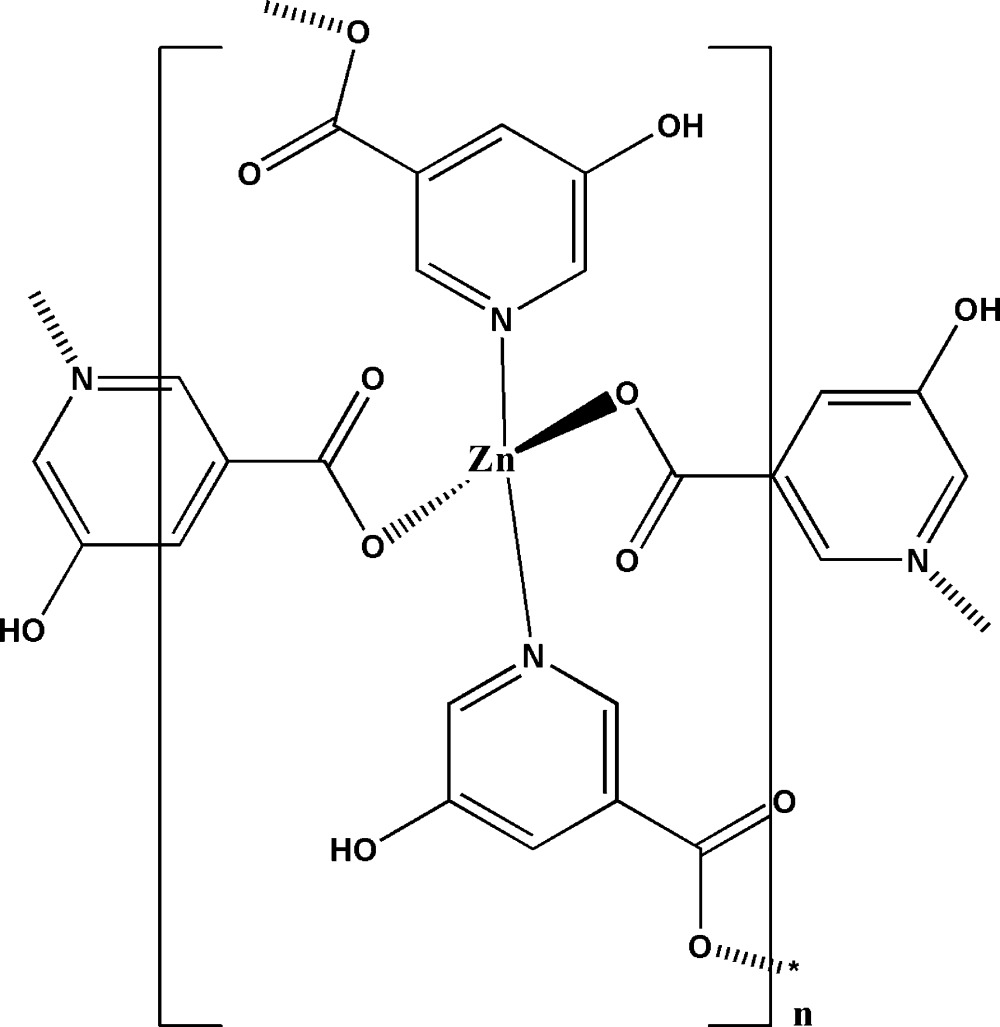


## Experimental   

### Crystal data  

[Zn(C_6_H_4_NO_3_)_2_]
*M* *_r_* = 341.57
Monoclinic, 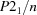

*a* = 9.4299 (6) Å
*b* = 10.5453 (7) Å
*c* = 12.6914 (8) Å
β = 104.640 (7)°
*V* = 1221.07 (14) Å^3^
*Z* = 4
Mo *K*α radiation
μ = 2.04 mm^−1^
*T* = 150 K
0.41 × 0.37 × 0.17 mm

### Data collection  

Bruker APEXII CCD diffractometer
Absorption correction: multi-scan (*SADABS*; Bruker, 2007 ▸) *T*_min_ = 0.488, *T*_max_ = 0.723
7054 measured reflections
2891 independent reflections
2397 reflections with *I* > 2σ(*I*)
*R*_int_ = 0.034

### Refinement  

*R*[*F* ^2^ > 2σ(*F* ^2^)] = 0.033
*wR*(*F* ^2^) = 0.075
*S* = 1.07
3345 reflections
192 parameters
H-atom parameters constrained
Δρ_max_ = 0.42 e Å^−3^
Δρ_min_ = −0.43 e Å^−3^

### 

Data collection: *APEX2* (Bruker, 2007 ▸); cell refinement: *SAINT* (Bruker, 2007 ▸); data reduction: *SAINT*; program(s) used to solve structure: *SHELXTL* (Sheldrick, 2008 ▸); program(s) used to refine structure: *SHELXTL*; molecular graphics: *DIAMOND* (Brandenburg, 2005 ▸); software used to prepare material for publication: *publCIF* (Westrip, 2010 ▸).

## Supplementary Material

Crystal structure: contains datablock(s) I, global. DOI: 10.1107/S2056989015000249/wm5106sup1.cif

Structure factors: contains datablock(s) I. DOI: 10.1107/S2056989015000249/wm5106Isup2.hkl

**Fig. 1.**
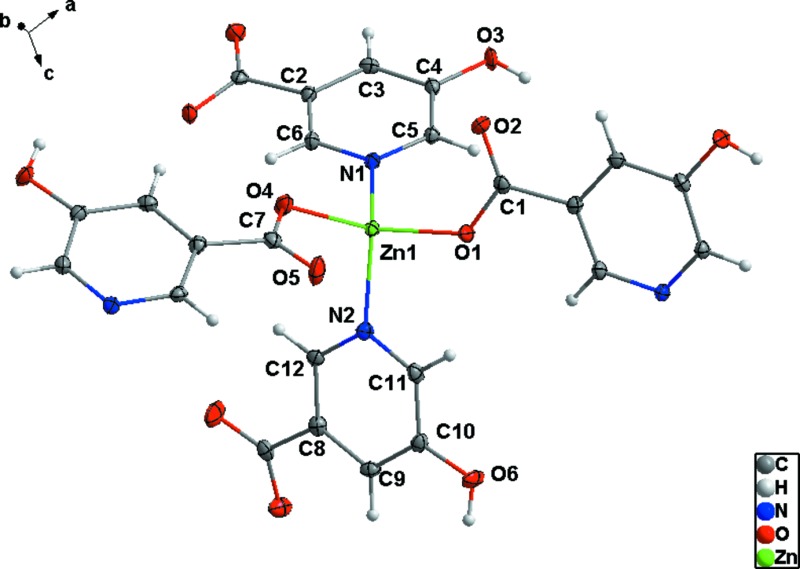
The coordination environment of the ZnII ion in the title compound, showing displacement ellipsoids at the 50% probability level.

II . DOI: 10.1107/S2056989015000249/wm5106fig1.tif
The coordination environment of the Zn^II^ ion in the title compound, showing displacement ellipsoids at the 50% probability level.

**Fig. 2.**
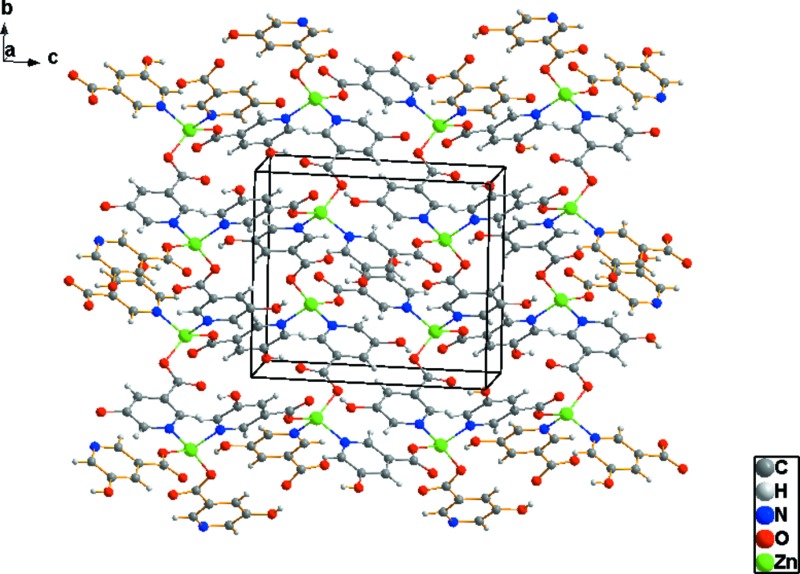
The packing in the structure of [Zn(C6H4O3N)2]n, showing the polymeric character of the title compound.

6 4 3 2 n . DOI: 10.1107/S2056989015000249/wm5106fig2.tif
The packing in the structure of [Zn(C_6_H_4_O_3_N)_2_]_n_, showing the polymeric character of the title compound.

CCDC reference: 1042412

Additional supporting information:  crystallographic information; 3D view; checkCIF report

## Figures and Tables

**Table 1:** Hydrogen-bond geometry (, )

*D*H*A*	*D*H	H*A*	*D* *A*	*D*H*A*
O3H3*A*O5^i^	0.82	1.88	2.697(2)	175
O6H6*A*O2^ii^	0.82	1.83	2.651(3)	174

Symmetry codes: (i) 
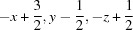
; (ii) 
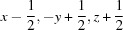
.

## supplementary crystallographic information

### S1. Experimental

A mixture of zinc nitrate, 5-hydroxynicotinic acid, malonic acid and water in a mole ratio of *ca* 1:2:1:550 was added to a 25 ml Teflon-lined cup, and the pH value of the mixture was adjusted to 6.5 by 5%_wt_ ammonia/water at room temperature. The Teflon-lined cup was sealed in a stainless steel vessel and heated to 443 K, kept at that temperature for 3 days, and then slowly cooled to room temperature at a rate of 5 K per hour. Yellow block-like crystals of the title compound were obtained. The yield was about 55%. Elemental anal. calc. for C_12_H_8_N_2_O_6_Zn (341.57): C 28.60, H 2.79, N, 3.28. Found: C 28.65, H 2.81, N, 3.13. IR (cm^-1^, KBr): 3454(*s*), 3104(*m*), 1856(w), 1632(*s*), 1586(*s*), 1487(*m*), 1432(*m*), 1400(*s*), 1302(w), 1279(*s*), 1239(*m*), 1158(w), 1119(w), 1026(*s*), 968(w), 936(*s*), 898(*s*), 822(*s*), 787(*s*), 732(*m*), 710(*m*), 687(*s*), 594(*m*), 539(*m*), 485(*m*), 453(w).

### S2. Refinement

Hydrogen atoms bonded to C atoms of the 5-hydroxynicotinate anions were positioned geometrically and refined as riding atoms, with C—H = 0.93 Å and *U*_iso_(H) = 1.2*U*_eq_(C). H atoms of the hydroxy functions were found from difference maps and were included in the refinement as riding atoms, with O—H = 0.82 Å and *U*_iso_(H) = 1.5*U*_eq_(O).

### Crystal data

**Table:**

[Zn(C_6_H_4_NO_3_)_2_]	*Z* = 4
*M**_r_* = 341.57	*F*(000) = 688
Monoclinic, *P*2_1_/*n*	*D*_x_ = 1.858 Mg m^−^^3^
Hall symbol: -P 2yn	Mo *K*α radiation, λ = 0.71073 Å
*a* = 9.4299 (6) Å	θ = 3.0–29.1°
*b* = 10.5453 (7) Å	µ = 2.04 mm^−^^1^
*c* = 12.6914 (8) Å	*T* = 150 K
β = 104.640 (7)°	Block, yellow
*V* = 1221.07 (14) Å^3^	0.41 × 0.37 × 0.17 mm

### Data collection

**Table:**

Bruker APEXII CCD diffractometer	2891 independent reflections
Radiation source: fine-focus sealed tube	2397 reflections with *I* > 2σ(*I*)
Graphite monochromator	*R*_int_ = 0.034
φ and ω scans	θ_max_ = 29.3°, θ_min_ = 2.4°
Absorption correction: multi-scan (*SADABS*; Bruker, 2007)	*h* = −12→12
*T*_min_ = 0.488, *T*_max_ = 0.723	*k* = −14→12
7054 measured reflections	*l* = −17→17

### Refinement

**Table:**

Refinement on *F*^2^	Primary atom site location: structure-invariant direct methods
Least-squares matrix: full	Secondary atom site location: difference Fourier map
*R*[*F*^2^ > 2σ(*F*^2^)] = 0.033	Hydrogen site location: inferred from neighbouring sites
*wR*(*F*^2^) = 0.075	H-atom parameters constrained
*S* = 1.07	*w* = 1/[σ^2^(*F*_o_^2^) + (0.0271*P*)^2^ + 0.5488*P*] where *P* = (*F*_o_^2^ + 2*F*_c_^2^)/3
3345 reflections	(Δ/σ)_max_ = 0.001
192 parameters	Δρ_max_ = 0.42 e Å^−^^3^
0 restraints	Δρ_min_ = −0.43 e Å^−^^3^

### Special details

**Table:**

Geometry. All e.s.d.'s (except the e.s.d. in the dihedral angle between two l.s. planes) are estimated using the full covariance matrix. The cell e.s.d.'s are taken into account individually in the estimation of e.s.d.'s in distances, angles and torsion angles; correlations between e.s.d.'s in cell parameters are only used when they are defined by crystal symmetry. An approximate (isotropic) treatment of cell e.s.d.'s is used for estimating e.s.d.'s involving l.s. planes.
Refinement. Refinement of *F*^2^ against ALL reflections. The weighted *R*-factor *wR* and goodness of fit *S* are based on *F*^2^, conventional *R*-factors *R* are based on *F*, with *F* set to zero for negative *F*^2^. The threshold expression of *F*^2^ > σ(*F*^2^) is used only for calculating *R*-factors(gt) *etc*. and is not relevant to the choice of reflections for refinement. *R*-factors based on *F*^2^ are statistically about twice as large as those based on *F*, and *R*- factors based on ALL data will be even larger.

### Fractional atomic coordinates and isotropic or equivalent isotropic displacement parameters (Å^2^)

**Table:**

	*x*	*y*	*z*	*U*_iso_*/*U*_eq_	
C1	0.8597 (2)	0.3757 (2)	0.30816 (18)	0.0111 (5)	
C2	0.4890 (2)	0.1376 (2)	−0.09332 (18)	0.0104 (5)	
C3	0.6200 (2)	0.0759 (2)	−0.08906 (19)	0.0118 (5)	
H3	0.6325	0.0305	−0.1489	0.014*	
C4	0.7328 (2)	0.0828 (2)	0.00604 (19)	0.0114 (5)	
C5	0.7092 (2)	0.1514 (2)	0.09361 (19)	0.0114 (5)	
H5	0.7828	0.1540	0.1581	0.014*	
C6	0.4744 (2)	0.2077 (2)	−0.00397 (19)	0.0123 (5)	
H6	0.3875	0.2512	−0.0078	0.015*	
C7	0.3884 (2)	0.5320 (2)	0.19736 (19)	0.0135 (5)	
C8	0.2504 (2)	0.1059 (2)	0.34892 (19)	0.0121 (5)	
C9	0.3220 (3)	0.0977 (2)	0.45873 (19)	0.0122 (5)	
H9	0.2824	0.0509	0.5065	0.015*	
C10	0.4545 (3)	0.1615 (2)	0.49542 (19)	0.0126 (5)	
C11	0.5125 (3)	0.2258 (2)	0.42054 (19)	0.0131 (5)	
H11	0.6029	0.2656	0.4450	0.016*	
C12	0.3118 (3)	0.1758 (2)	0.27914 (19)	0.0117 (5)	
H12	0.2610	0.1836	0.2064	0.014*	
N1	0.5831 (2)	0.21429 (19)	0.08815 (15)	0.0108 (4)	
N2	0.4436 (2)	0.2330 (2)	0.31413 (15)	0.0122 (4)	
O1	0.74133 (17)	0.32799 (17)	0.32074 (13)	0.0154 (4)	
O2	0.87405 (18)	0.43130 (18)	0.22547 (13)	0.0179 (4)	
O3	0.85858 (17)	0.02136 (18)	0.00882 (14)	0.0168 (4)	
H3A	0.9134	0.0264	0.0702	0.025*	
O4	0.41994 (18)	0.44819 (17)	0.13440 (13)	0.0178 (4)	
O5	0.45953 (19)	0.55268 (19)	0.29152 (14)	0.0238 (5)	
O6	0.53504 (19)	0.16272 (18)	0.60036 (13)	0.0188 (4)	
H6A	0.4885	0.1288	0.6391	0.028*	
Zn1	0.55375 (3)	0.32108 (3)	0.21439 (2)	0.01009 (9)	

### Atomic displacement parameters (Å^2^)

**Table:**

	*U*^11^	*U*^22^	*U*^33^	*U*^12^	*U*^13^	*U*^23^
C1	0.0105 (10)	0.0118 (12)	0.0110 (11)	0.0024 (10)	0.0028 (9)	−0.0023 (9)
C2	0.0105 (10)	0.0102 (12)	0.0101 (11)	0.0001 (10)	0.0016 (9)	0.0015 (9)
C3	0.0130 (11)	0.0122 (13)	0.0113 (11)	−0.0013 (10)	0.0050 (9)	−0.0009 (10)
C4	0.0083 (10)	0.0120 (13)	0.0139 (12)	0.0006 (10)	0.0026 (9)	0.0016 (10)
C5	0.0097 (10)	0.0129 (13)	0.0104 (11)	0.0005 (10)	0.0006 (9)	0.0001 (9)
C6	0.0089 (11)	0.0160 (13)	0.0111 (12)	0.0011 (10)	0.0008 (9)	0.0005 (9)
C7	0.0107 (11)	0.0143 (13)	0.0158 (13)	−0.0011 (10)	0.0039 (10)	0.0019 (10)
C8	0.0122 (11)	0.0104 (12)	0.0132 (12)	0.0004 (10)	0.0021 (9)	−0.0021 (9)
C9	0.0154 (11)	0.0100 (12)	0.0122 (11)	−0.0003 (10)	0.0058 (9)	0.0001 (9)
C10	0.0129 (11)	0.0143 (13)	0.0097 (11)	0.0017 (10)	0.0012 (9)	−0.0017 (9)
C11	0.0100 (11)	0.0159 (13)	0.0128 (12)	−0.0013 (10)	0.0016 (10)	−0.0029 (10)
C12	0.0123 (11)	0.0128 (13)	0.0102 (11)	0.0026 (10)	0.0034 (9)	−0.0011 (9)
N1	0.0097 (9)	0.0127 (11)	0.0093 (9)	0.0001 (8)	0.0011 (8)	−0.0007 (8)
N2	0.0121 (9)	0.0131 (11)	0.0113 (10)	−0.0014 (9)	0.0028 (8)	−0.0009 (8)
O1	0.0096 (8)	0.0246 (10)	0.0109 (8)	−0.0041 (7)	0.0006 (7)	0.0016 (7)
O2	0.0157 (8)	0.0280 (11)	0.0097 (8)	0.0012 (8)	0.0025 (7)	0.0045 (7)
O3	0.0092 (8)	0.0250 (10)	0.0144 (9)	0.0079 (8)	−0.0002 (7)	−0.0027 (8)
O4	0.0190 (9)	0.0203 (10)	0.0136 (9)	0.0090 (8)	0.0029 (7)	0.0021 (7)
O5	0.0191 (9)	0.0302 (12)	0.0164 (10)	0.0070 (8)	−0.0060 (8)	−0.0029 (8)
O6	0.0184 (9)	0.0295 (12)	0.0071 (8)	−0.0073 (8)	0.0008 (7)	−0.0013 (7)
Zn1	0.00810 (14)	0.01335 (17)	0.00817 (15)	0.00045 (11)	0.00084 (10)	−0.00090 (11)

### Geometric parameters (Å, º)

**Table:**

C1—O2	1.239 (3)	C8—C12	1.387 (3)
C1—O1	1.270 (3)	C8—C9	1.389 (3)
C1—C2^i^	1.516 (3)	C8—C7^iv^	1.509 (3)
C2—C3	1.385 (3)	C9—C10	1.392 (3)
C2—C6	1.390 (3)	C9—H9	0.9300
C2—C1^ii^	1.516 (3)	C10—O6	1.356 (3)
C3—C4	1.395 (3)	C10—C11	1.387 (3)
C3—H3	0.9300	C11—N2	1.345 (3)
C4—O3	1.344 (3)	C11—H11	0.9300
C4—C5	1.390 (3)	C12—N2	1.351 (3)
C5—N1	1.348 (3)	C12—H12	0.9300
C5—H5	0.9300	N1—Zn1	2.034 (2)
C6—N1	1.348 (3)	N2—Zn1	2.052 (2)
C6—H6	0.9300	O1—Zn1	1.9364 (15)
C7—O5	1.233 (3)	O3—H3A	0.8200
C7—O4	1.276 (3)	O4—Zn1	1.9421 (17)
C7—C8^iii^	1.509 (3)	O6—H6A	0.8200
			
O2—C1—O1	125.5 (2)	C10—C9—H9	120.9
O2—C1—C2^i^	120.3 (2)	O6—C10—C11	116.6 (2)
O1—C1—C2^i^	114.1 (2)	O6—C10—C9	124.5 (2)
C3—C2—C6	119.3 (2)	C11—C10—C9	118.9 (2)
C3—C2—C1^ii^	120.8 (2)	N2—C11—C10	122.8 (2)
C6—C2—C1^ii^	119.8 (2)	N2—C11—H11	118.6
C2—C3—C4	119.1 (2)	C10—C11—H11	118.6
C2—C3—H3	120.4	N2—C12—C8	121.7 (2)
C4—C3—H3	120.4	N2—C12—H12	119.2
O3—C4—C5	123.2 (2)	C8—C12—H12	119.2
O3—C4—C3	118.2 (2)	C5—N1—C6	119.1 (2)
C5—C4—C3	118.5 (2)	C5—N1—Zn1	121.74 (15)
N1—C5—C4	122.2 (2)	C6—N1—Zn1	119.12 (16)
N1—C5—H5	118.9	C11—N2—C12	118.4 (2)
C4—C5—H5	118.9	C11—N2—Zn1	116.97 (15)
N1—C6—C2	121.7 (2)	C12—N2—Zn1	124.49 (16)
N1—C6—H6	119.2	C1—O1—Zn1	127.16 (15)
C2—C6—H6	119.2	C4—O3—H3A	109.5
O5—C7—O4	125.0 (2)	C7—O4—Zn1	111.97 (15)
O5—C7—C8^iii^	119.4 (2)	C10—O6—H6A	109.5
O4—C7—C8^iii^	115.5 (2)	O1—Zn1—O4	134.17 (8)
C12—C8—C9	119.9 (2)	O1—Zn1—N1	106.71 (7)
C12—C8—C7^iv^	119.2 (2)	O4—Zn1—N1	99.80 (8)
C9—C8—C7^iv^	120.8 (2)	O1—Zn1—N2	95.90 (7)
C8—C9—C10	118.2 (2)	O4—Zn1—N2	105.76 (8)
C8—C9—H9	120.9	N1—Zn1—N2	115.24 (8)

Symmetry codes: (i) *x*+1/2, −*y*+1/2, *z*+1/2; (ii) *x*−1/2, −*y*+1/2, *z*−1/2; (iii) −*x*+1/2, *y*+1/2, −*z*+1/2; (iv) −*x*+1/2, *y*−1/2, −*z*+1/2.

### Hydrogen-bond geometry (Å, º)

**Table:**

*D*—H···*A*	*D*—H	H···*A*	*D*···*A*	*D*—H···*A*
O3—H3*A*···O5^v^	0.82	1.88	2.697 (2)	175
O6—H6*A*···O2^vi^	0.82	1.83	2.651 (3)	174

Symmetry codes: (v) −*x*+3/2, *y*−1/2, −*z*+1/2; (vi) *x*−1/2, −*y*+1/2, *z*+1/2.

## References

[bb1] Brandenburg, K. (2005). *DIAMOND*. Crystal Impact GbR, Bonn, Germany.

[bb2] Bruker (2007). *APEX2*, *SAINT* and *SADABS*. Bruker AXS Inc. Madison, Wisconsin, USA.

[bb4] Jiang, M.-X. & Feng, Y.-L. (2008). *Acta Cryst.* E**64**, m1517. 10.1107/S1600536808035903 PMC2960042 21581135

[bb5] Mi, J.-L., Huang, J. & Chen, H.-J. (2012). *Acta Cryst.* E**68**, m1146–m1147. 10.1107/S1600536812032916 PMC3435576 22969449

[bb6] Sheldrick, G. M. (2008). *Acta Cryst* A**64**, 112–122. 10.1107/S0108767307043930 18156677

[bb7] Westrip, S. P. (2010). *J. Appl. Cryst.* **43**, 920–925.

[bb8] Xu, S.-S., Mi, J.-L. & Chen, H.-J. (2013). *Acta Cryst.* E**69**, m294–m295. 10.1107/S1600536813011057 PMC3647824 23723790

[bb9] Yang, J., Chen, H. J. & Tsz, H. L. (2010). *Inorg. Chem. Commun* **10**, 1016–1019.

[bb3] Zhang, J., Chen, H.-J. & Huang, J. (2011). *Chin. J. Struct. Chem.* **30**, 1069–1073.

[bb10] Zhang, J., Huang, J., Yang, J. & Chen, H.-J. (2012). *Inorg. Chem. Commun.* **17**, 163–168.

