# Supplementary figures and images for: Crystal structure of poly[bis­(μ2-5-hydroxy­nicotinato-κ2 N:O 3)zinc]
Source: Acta Crystallogr E Crystallogr Commun. 2015 Jan 14;71(Pt 2):m23. doi: 10.1107/S2056989015000249 (PMC4384582; doi:10.1107/S2056989015000249)

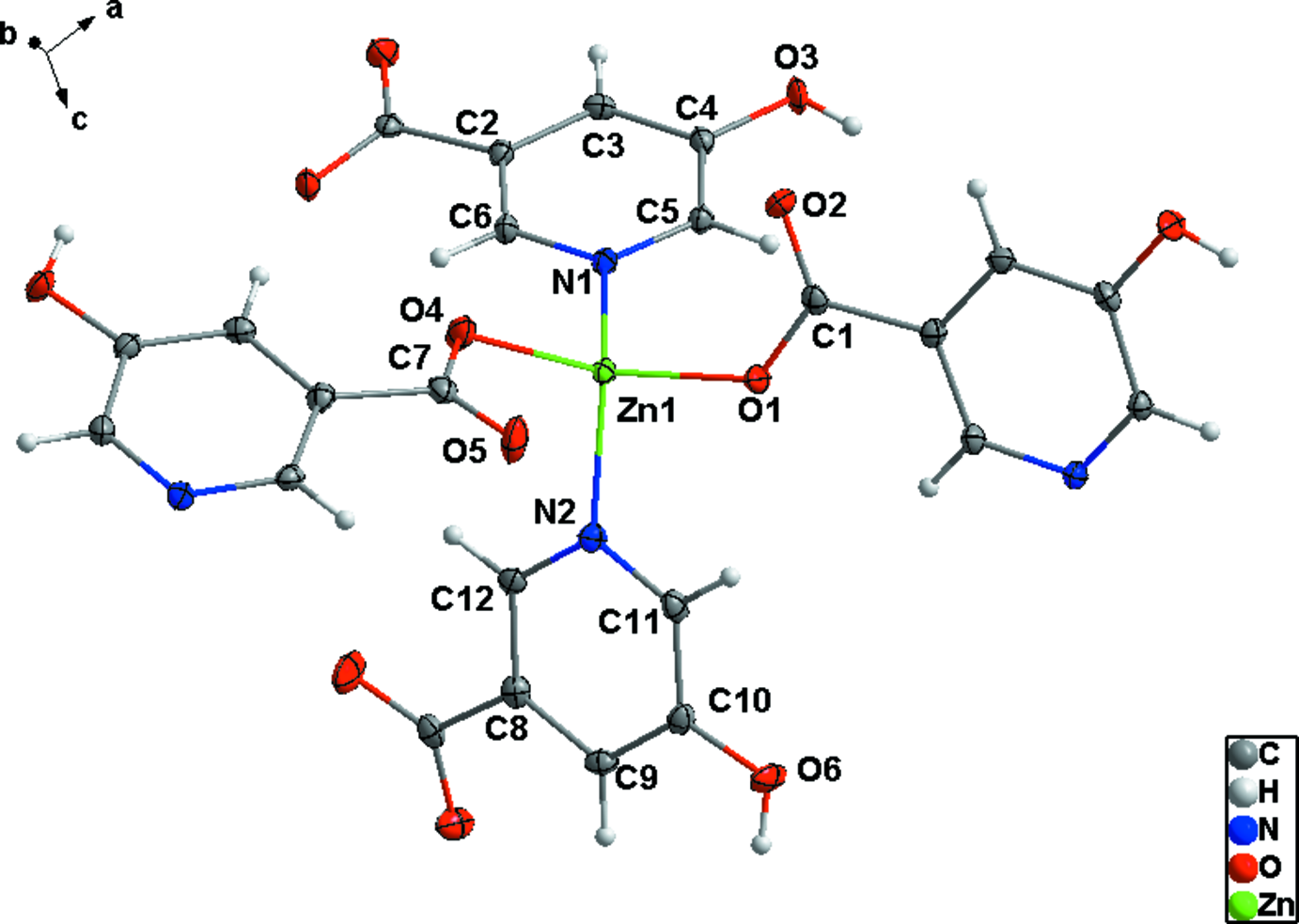

Supplement: Supplementary file 3 [file e-71-00m23-fig1.tif]

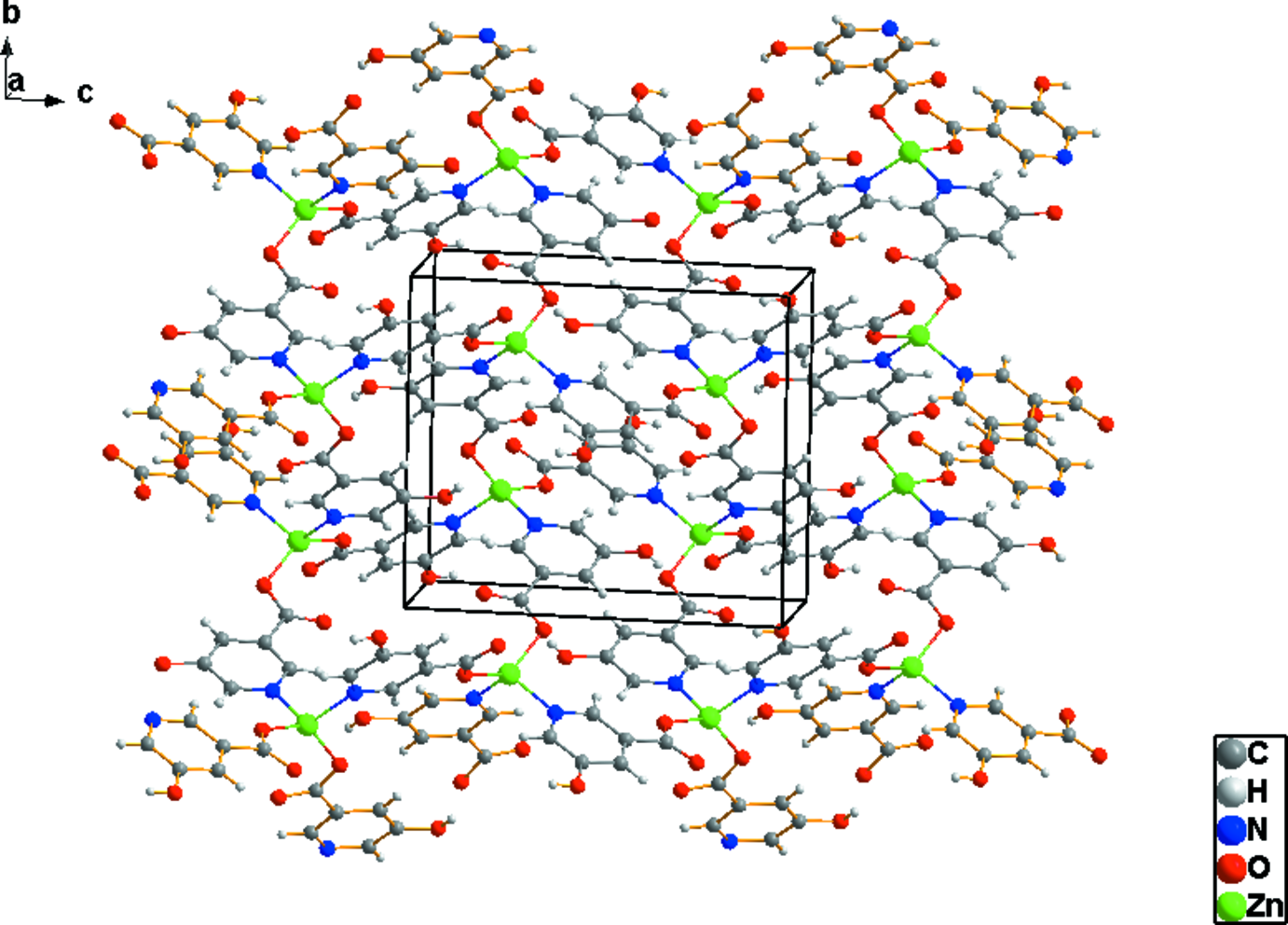

Supplement: Supplementary file 4 [file e-71-00m23-fig2.tif]
